# Beta 1 integrin predicts survival in breast cancer: a clinicopathological and immunohistochemical study

**DOI:** 10.1186/1746-1596-7-104

**Published:** 2012-08-16

**Authors:** Petra Barros dos Santos, Juliana S Zanetti, Alfredo Ribeiro-Silva, Eduardo IC Beltrão

**Affiliations:** 1 Department of Pathology, Laboratory of Immunopathology Keizo Asami, Federal University of Pernambuco, Avenida Professor Moraes Rêgo S/N, 50670-901, Recife, Pernambuco, Brazil; 2 Department of Pathology, Ribeirão Preto Medical School, University of São Paulo, Ribeirão Preto, São Paulo, 14049-900, Brazil; 3 Department of Biochemistry, Federal University of Pernambuco, Recife, Pernambuco, 50670-901, Brazil

**Keywords:** β1 integrin, Tissue microarray, Immunohistochemistry, Prognosis

## Abstract

**Background:**

The main focus of several studies concerned with cancer progression and metastasis is to analyze the mechanisms that allow cancer cells to interact and quickly adapt with their environment. Integrins, a family of transmembrane glycoproteins, play a major role in invasive and metastatic processes. Integrins are involved in cell adhesion in both cell-extracellular matrix and cell-cell interactions, and particularly, β1 integrin is involved in proliferation and differentiation of cells in the development of epithelial tissues. This work aimed to investigate the putative role of β1 integrin expression on survival and metastasis in patients with breast invasive ductal carcinoma (IDC). In addition, we compared the expression of β1 integrin in patients with ductal carcinoma in situ (DCIS).

**Methods:**

Through tissue microarray (TMA) slides containing 225 samples of IDC and 67 samples of DCIS, β1 integrin expression was related with several immunohistochemical markers and clinicopathologic features of prognostic significance.

**Results:**

β1 integrin was overexpressed in 32.8% of IDC. In IDC, β1 integrin was related with HER-2 (p = 0.019) and VEGF (p = 0.011) expression and it had a significant relationship with metastasis and death (p = 0.001 and p = 0.05, respectively). Kaplan-Meier survival analysis showed that the overexpression of this protein is very significant (p = 0.002) in specific survival (number of months between diagnosis and death caused by the disease). There were no correlation between IDC and DCIS (p = 0.559) regarding β1 integrin expression.

**Conclusions:**

Considering that the expression of β1 integrin in breast cancer remains controversial, specially its relation with survival of patients, our findings provide further evidence that β1 integrin can be a marker of poor prognosis in breast cancer.

**Virtual slides:**

The virtual slide(s) for this article can be found here: http://www.diagnosticpathology.diagnomx.eu/vs/6652215267393871

## Background

Integrins are a family of transmembrane receptors. They are heterodimers composed by two subunits, α and β, which are non-covalently linked and depend of divalent cations [1]. A total of 18α and 8β subunits have been identified. These subunits combine to form 24 distinct heterodimers, however splice variants have been identified for some subunits and the number can reach at least 100 types [2,3]. These molecules can mediate intra and extracellular signals involved in the organization of cells, tissues and organs during development. Moreover they can directly or indirectly influence on many aspects of cell behavior, such adhesion, migration and proliferation. They also play a significant role in signal transduction events, such as gene expression and regulation of cell apoptosis [4,5].

Invasive breast carcinoma is associated with a high mortality rate due to invasion to the lymph nodes, adjacent tissues and also due to metastasis. Invasive ductal carcinoma (IDC) is the most common histological type, accounting for approximately 40–75% of all cases. IDC has a relatively poor prognosis with 35–50% 10-year survival rate [6,7]. Traditionally, as also verified in several other solid tumors, peritumoral lymphatic and blood invasion are the main factor related to the presence of lymph node metastasis and, in breast cancer, they are more closely related to tumor size and histological grade and it is known that integrins are closely related to these processes [8,9].

β1 integrin is the mainly expressed integrin in normal cells and in tumor-associated cells, in which they control various developmental processes including angiogenesis, tumor progression and metastasis [10]. These integrins typically mediate adhesion of epithelial cells to the basement membrane. They may also contribute to cell survival of tumor cells by interacting with others molecules. β1 integrin usually activate cytokine receptors or growth factors receptors (FGRs) and as a result, the tumor growth and invasion probably depends on the crosstalk with certain integrins, FGRs and/or oncogenes in tumor cells and tumor-associated cells [11]. Based on these observations, β1 integrin has become a target of interest for immunotherapy in several types of cancers, including breast cancer [12]. In recent years, several β1 integrins antagonists have been studied [13,14].

Over the last years, the study of ductal carcinoma in situ (DCIS) associated with invasive carcinoma has helped to understand the mechanisms involved in disease progression from DCIS to IDC [15]. Many studies have been reported conflicting results about the role of integrins in cancer cells and also in the patients’ survival, and how these integrins may contribute to metastasis [16,17]. We aimed to evaluate the β1 integrin expression in IDC and its relationship with several biomarkers and clinicopathologic features of prognostic significance. In addition, we compared the pattern of β1 integrin expression in IDC and DCIS.

## Methods

### Casuistic

This study was approved by the local Research Ethics Committee. Formalin-fixed, paraffin-embedded samples of IDC and DCIS from 300 and 100 patients, respectively, diagnosed from 1994 to 2010, were randomly chosen from the archives of Department of Pathology-Ribeirão Preto Medical School, São Paulo University. The clinical data of these patients were retrieved from medical files, and included age, menopausal status, tumor size, metastasis to regional lymph nodes, recurrence, distant metastasis and death. Disease-specific survival, metastasis-free survival and disease-free survival were defined by Zanetti and colleagues [18].

For DCIS patients the following information was retrieved from clinical and pathological records: age, menstrual status, hormone receptors status (ER and PR), nuclear grade (low, intermediary and high), tumor size and if the tumor was multifocal or not.

### Tissue microarray (TMA)

All hematoxylin and eosin stained slides were reviewed by an experienced breast pathologist (ARS). Three tissue microarrays (TMA) paraffin blocks were constructed from the IDC cases (100 cases per TMA) and five TMA paraffin blocks were constructed containing 24 DCIS cases per TMA. For the construction of the IDC and DCIS TMAs, core biopsies of 1-mm diameter were punched from the selected regions of each donor paraffin blocks and arrayed into the TMA receptor block. One section of each was stained with hematoxylin and eosin to confirm the presence of the tumor by light microscopy. The three IDC’s TMAs contained a total of 300 samples; however, 75 cases had to be excluded because of insufficient amount of tumor in the core. In that way, the study was performed in the 225 remaining cases that had high quality tissue spot that could be read for β1 integrin and the others markers. DCIS’s TMAs contained a total of 100 samples but only 67 had enough tumor tissue at the core, which were included in the analysis.

### Immunohistochemistry

Sections (3 μm) were obtained from the TMAs paraffin blocks and immunohistochemical reactions were performed with the Mach 4 Universal Polymer Detection kit (Biocare Medical, CA, USA) following protocols that were described elsewhere [18,19]. β1 integrin was detected using clone 4B7R (1:100) and VEGF with clone A-20 (1:100), both from Santa Cruz, Palo Alto, USA. The other antibodies are from Novocastra, Newcastle upon Tyne, UK: p53 (1:50, clone DO-7), ER (1:100, clone 6 F11), PR (1:100, clone 16), HER-2 (1:100, clone CB11) and Ki67 (1:100, clone MM1). Normal liver samples were used as positive control for β1 integrin. IDC cases previously known to be positive for Ki67, ER, PR, p53, HER-2 were used as positive controls for each reaction. Negative controls were prepared omitting the primary antibody.

### Immunohistochemistry evaluation

β1 integrin cut-off was based according to Yao and colleagues [20] in which sample was scored based on the intensity of signal (0, 1+, 2+ 3+) and the percentage of positive cells (0 ≤ 10%, 1 = 10–25%, 2 = 25–50%, 3 ≥ 50%). For statistical analysis we used the same method established by Petricevic and colleagues [21] where the results were presented as a positive (strong positive staining 3+ and moderately positive staining 2+) or a negative (weak positive 1+ and negative staining 0) for tumor cells.

HER-2 was evaluated according to the ASCO/CAP HER2 guideline [22] and cases as 2+ were submitted to chromogenic in situ hybridization (CISH) [23]. Only HER-2 2+ cases in immunohistochemistry amplified on CISH were considered positive for statistical purposes. VEGF immunoscoring, according to Giatromanolaki and colleagues [24], was divided into two groups regarding the extent of positive staining: low/medium reactivity (0–69% positive cells) and high reactivity (70-100% positive cells).

For ER and PR we follow the recommendations of the ASCO/CAP ER/PR guidelines (2010) [25]. Ki67 was evaluated following Fountzilas and collegues [26] and cases were considered highly proliferative when more than 14% of neoplastic cells nuclei were positive. p53 were considered positive if more than 5% of the neoplastic cells showed nuclear staining [27].

### Chromogenic in situ hybridization (CISH)

Sections (3 μm) were cut from paraffin blocks of HER-2 cases with 2+ immunohistochemistry positivity. ZytoDot 2C SPEC HER2/CEN 17 probe kit (Zytovision, Bremerhaven, Germany) was used for the detection of the human HER-2 gene and alpha-satellites of chromosome 17 (CEN 17). Procedures were according to manufacturer’s instructions where two green (HER-2) and two red (CEN 17) signals were expected in a normal interphase nucleus. HER-2 was considered amplified when the HER-2/CEN 17 ratio was ≥ 2 for 60 cells [28]. Only 2+ biopsies by immunohistochemistry in which HER-2 was also amplified on CISH were considered positive.

### Statistical methods

Data analysis was performed with SPSS v19.0 (SPSS Inc., Woking, UK) with p <0.05 for significance. Relation between β1 integrin, VEGF, HER-2 and the routine laboratory markers and clinicopathologic features were analyzed by Fisher exact test (two variables) or Chi-square tests (three or more variables), and all tests were two-tailed. Univariable survival analysis (disease-specific survival, disease-free survival and metastasis-free survival) were made with the log rank test and all results were displayed in Kaplan–Meier. A Cox Proportional Hazards Model was performed to observe the independent prognostic value of immunoexpression of β1 integrin.

## Results

### Relationship between the expression of β1 integrin with clinicopathological features and biological markers

IDC cases with less than 50% of representative tumor area by hematoxilin eosin staining were excluded. Two hundred twenty five cases of IDC were then analyzed. The average age of patients included in this study was 55 years old (range 25–85 years).

There was no significant relation between the expression of β1 integrin with biomarkers such ER and PR. These results are shown in Table 1. β1 integrin positive tumors did not correlate with tumor size, pathologic stage, age, menstrual status, lymph node status and tumor grade but presented a close relation with death and metastasis (p = 0.001 and p = 0.05, respectively) as well as with HER-2 (p = 0.019) and VEGF (p = 0.011).

**Table 1:** Relationship between β1 integrin expression with clinicopathologic features and classical immunohistochemical markers in breast cancer, including VEGF

**Clinicopathologic Features and Immunohistochemical Markers**		**β1 Integrin**^**-**^		**β1 Integrin**^**+**^		***P*-value**
		**n (151)**	**(%)**	**n (74)**	**(%)**	
Age (years)	<50	65	43.0	27	36.5	0.213^a^
	>50	86	67.0	47	63.5	
Menstrual Status	Pre-menopausal	54	35.8	24	32.4	0.367^a^
	Post-menopausal	97	64.2	50	67.6	
Size (mm)	<20	49	32.5	19	25.7	0.442^b^
	20-50	55	36.4	33	44.6	
	>50	47	31.1	22	29.7	
Tumoral Grade	I	57	37.7	25	33.8	0.845^b^
	II	71	47.0	37	50.0	
	III	23	15.2	12	16.2	
Lymph node Status	Negative	75	49.7	33	44.6	0.283^a^
	Positive	76	50.3	41	55.4	
Clinical Stage	I	22	14.6	10	13.5	0.677^b^
	II	72	47.7	30	40.5	
	III	49	32.5	30	40.5	
	IV	8	5.3	4	5.4	
Death	No	116	76.8	40	54.1	**0.001**^a^
	Yes	35	23.2	34	45.9	
Metastasis	No	115	76.2	48	64.9	**0.05**^a^
	Yes	36	23.8	26	35.1	
ER	Negative	48	31.8	21	28.4	0.359^a^
	Positive	103	68.2	53	71.6	
PR	Negative	64	42.4	33	44.6	0.431^a^
	Positive	87	57.6	41	55.4	
p53	Negative	114	75.5	51	68.9	0.187^a^
	Positive	37	24.5	23	31.1	
Ki-67	Negative	107	70.9	54	73.0	0.435^a^
	Positive	44	29.1	20	27.0	
HER2	Negative	126	83.4	52	70.3	**0.019**^a^
	Positive	25	16.6	22	29.7	
VEGF	Negative	114	75.5	66	89.2	**0.011**^a^
	Positive	37	24.5	8	10.8	

^a^Fisher’s exact test; ^b^Chi-square test.

### Survival analysis

**Figure 1:**
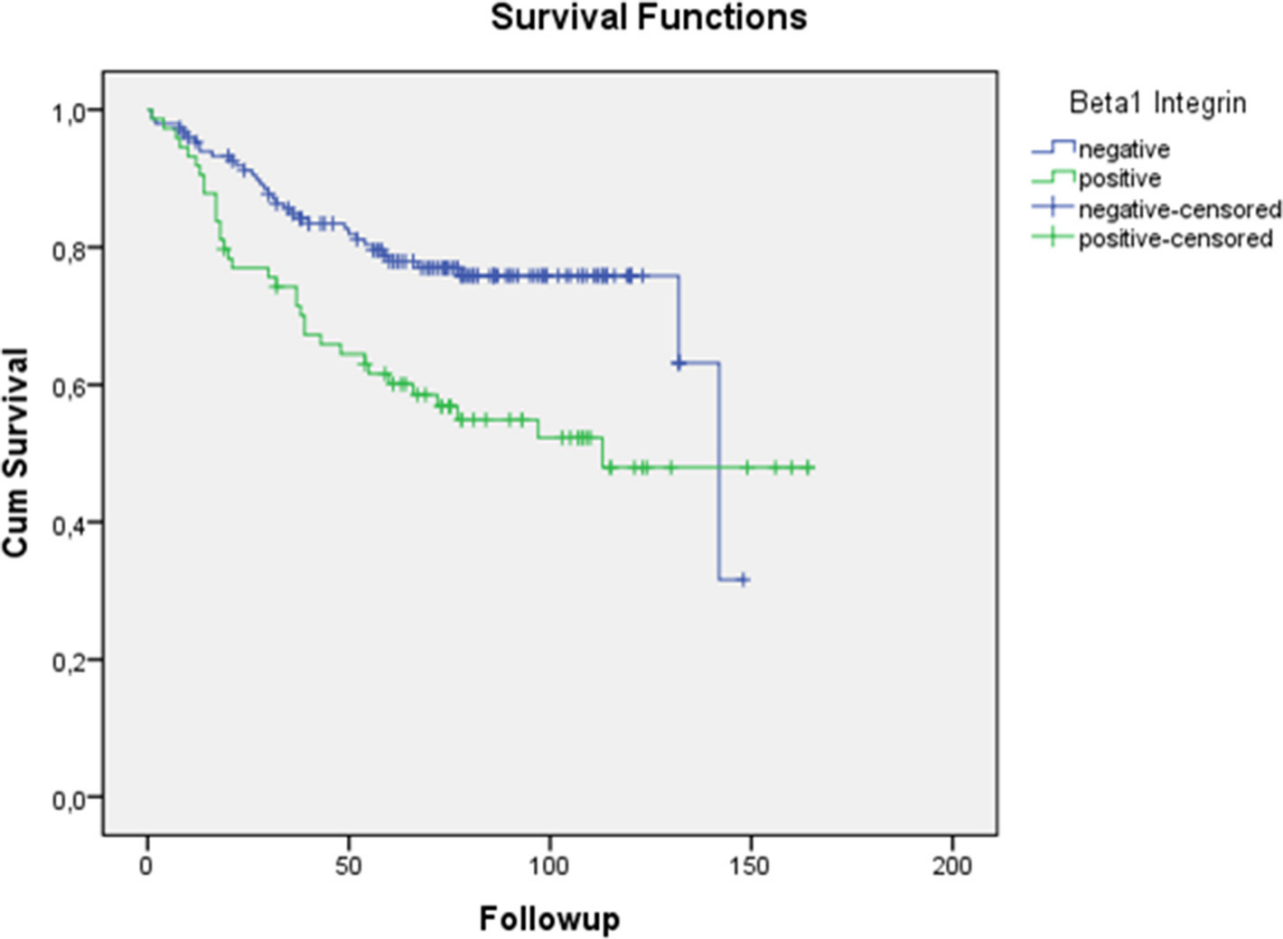
**Univariate analysis: the prognostic impact of β1 integrin status on disease-specific survival of breast cancer patients (p = 0.002).** (Using Kaplan-Meier table followed by log-rank test).

**Figure 2:**
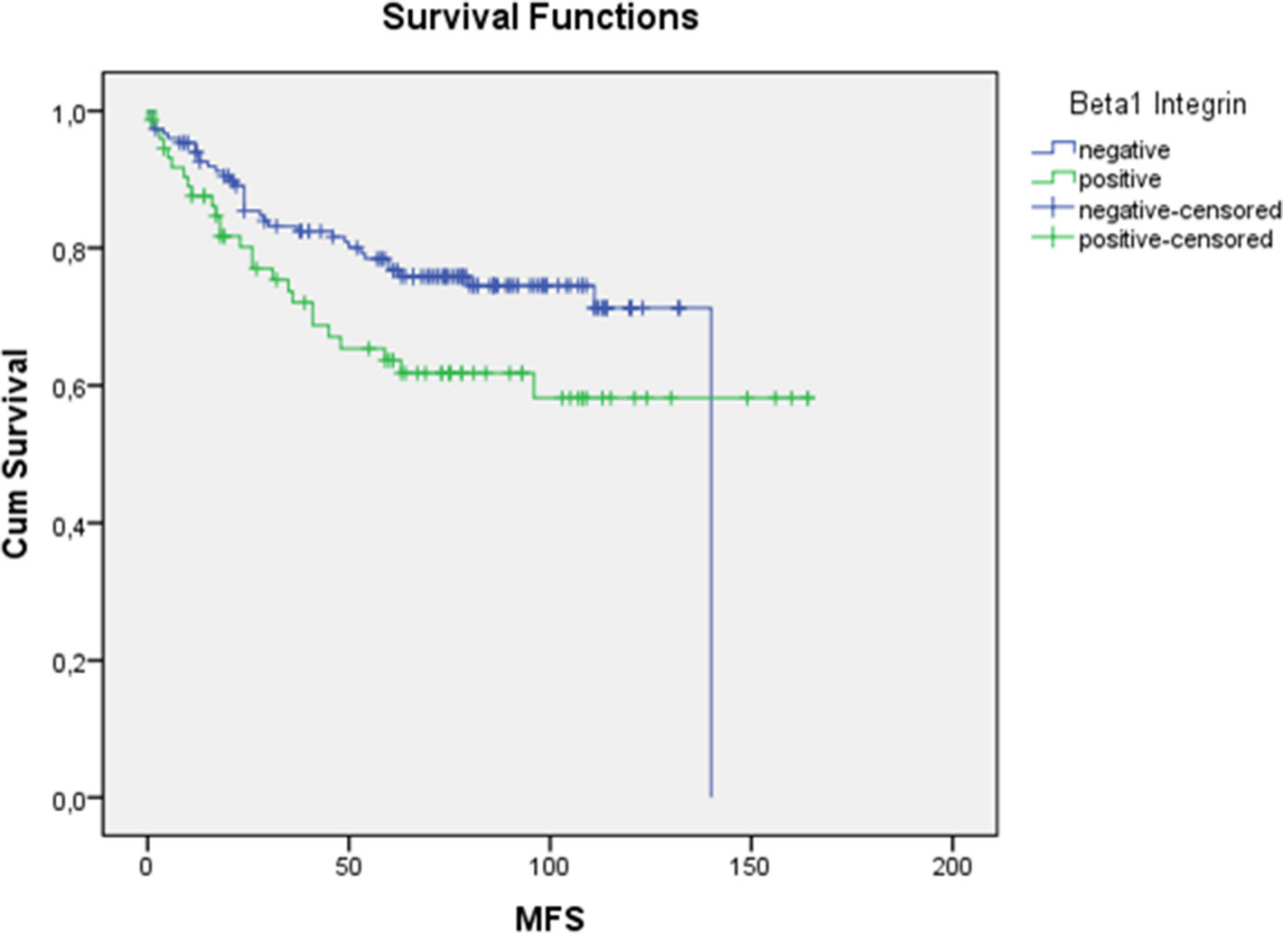
**Kaplan-Meier survival curves: comparison of the expression of β1 integrin (green solid line) to negativity (blue solid line) in metastasis-free survival (MFS) demonstrating the non-significant (p = 0.061) but clearly divergent curves.** (Using Kaplan-Meier table followed by log-rank test).

**Figure 3:**
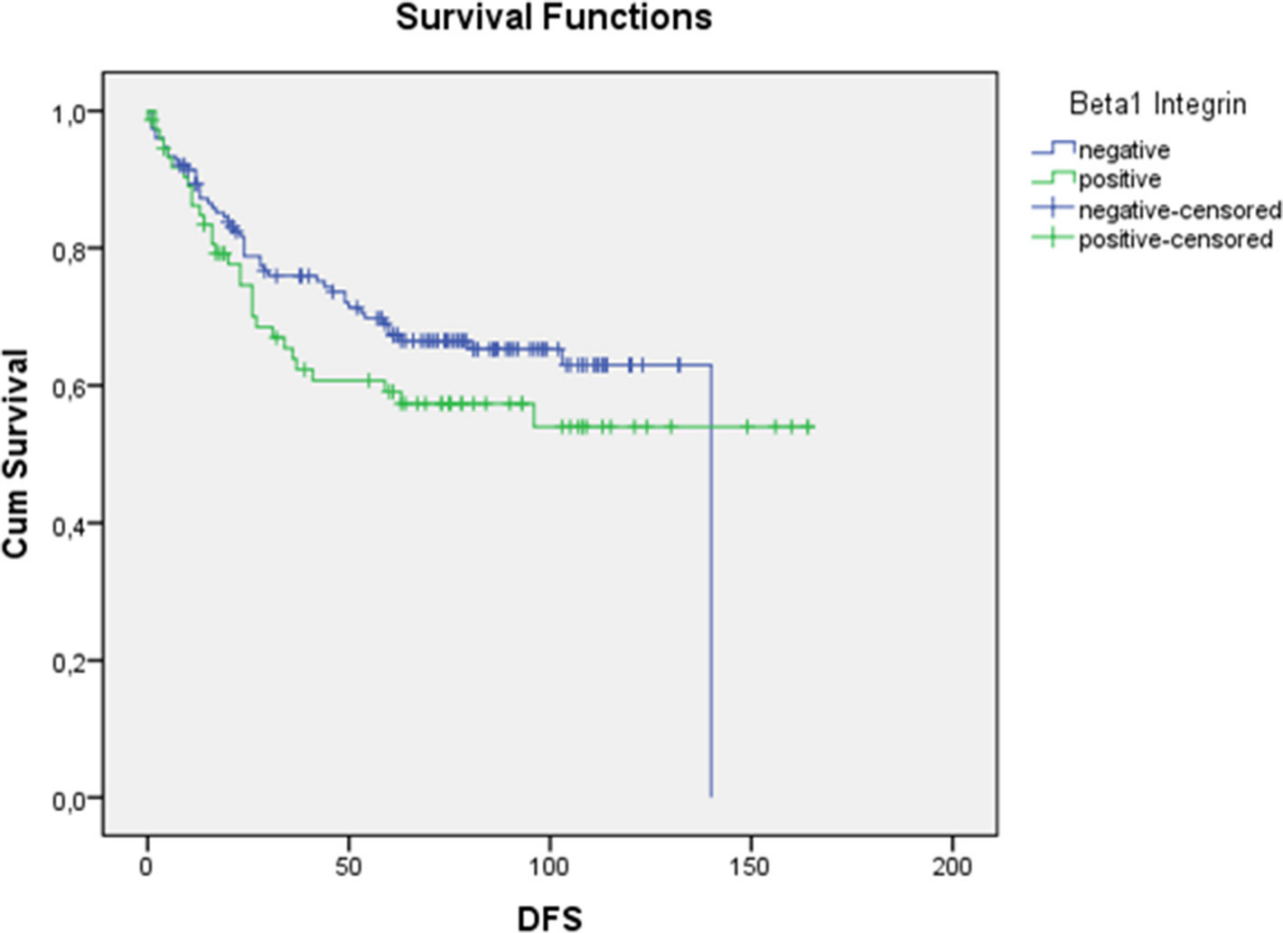
**Lack of significant difference (p = 0.252) in the curves for presence (green solid line) or absence (blue solid line) of expression for β1 subunit in disease free-survival (DFS).** (Using Kaplan-Meier table followed by log-rank test.)

Chi-square test for β1 integrin and correlation for death and metastasis showed that 35 patients died and that 34 out of these had β1 integrin superexpression and that 36 patients developed metastasis and that 26 out of them had β1 integrin superexpression. The Kaplan-Meier test was used to estimate the survival time of patients that expressed or not β1 integrin during the research time (maximum 164 months). Our results indicate that the expression of β1 integrin has an impact in disease-specific survival (number of months from diagnosis to the time of death due to breast cancer) with p = 0.002 (Figure 1). For metastasis-free survival (Figure 2) and disease-free survival (Figure 3) no significant relation was observed (p = 0.061 and p = 0,252, respectively).

A Cox Proportional Hazards Model was performed to observe the independent prognostic value of β1 integrin expression with several prognostic factors such menopausal status, clinical stage, Bloom-Richardson, recurrence, but no correlation was found; data not shown.

### IDC x DCIS

**Figure 4:**
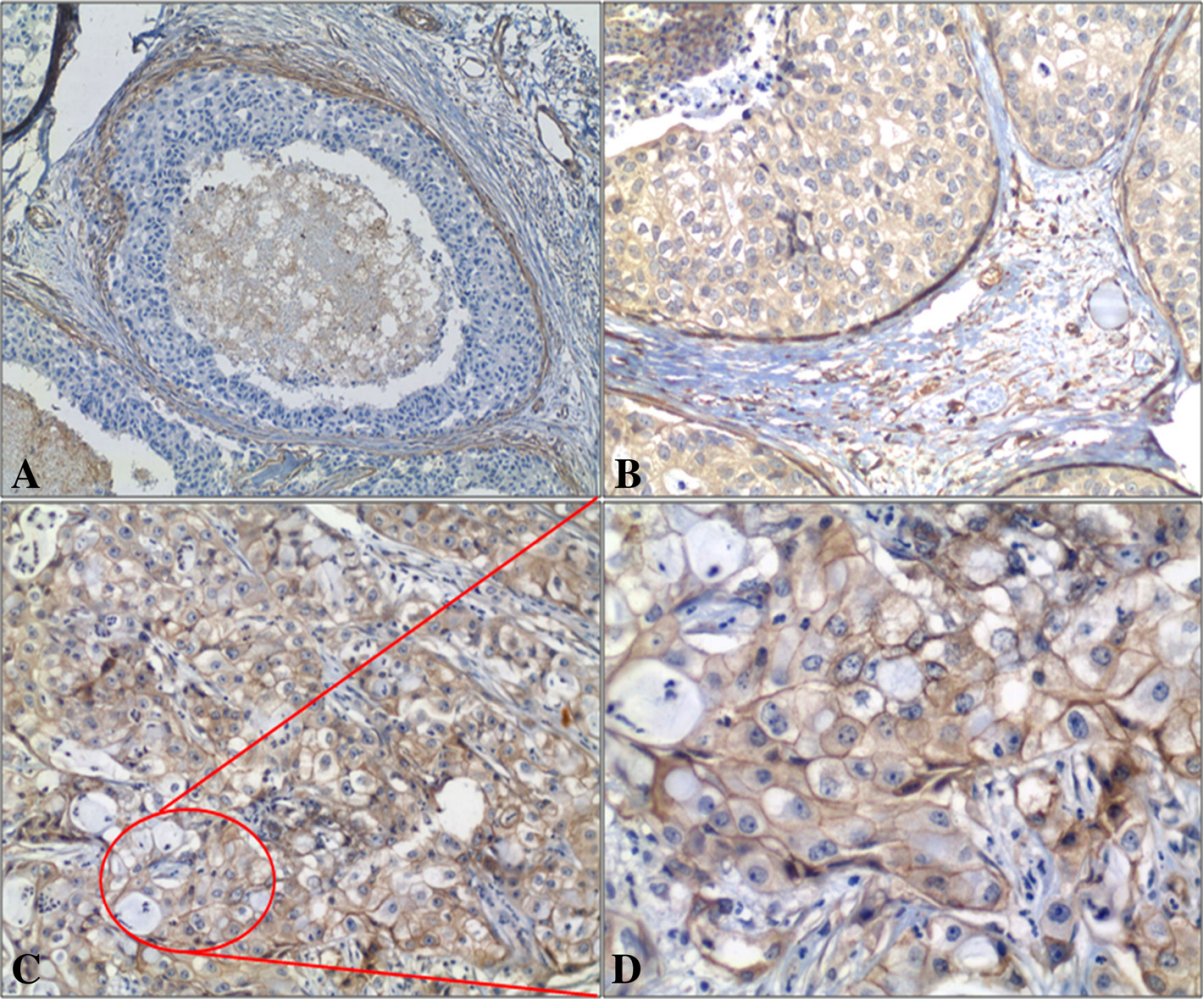
**Immunohistochemistry for β1 integrin in Invasive and In situ Breast Carcinoma.** DCIS: (**A**) negative (100×) and (**B**) positive (200×); IDC: (**C**) positive (200x) and (**D**) detail with 400x of the circled area. β1 integrin can be found both in cytoplasm and plasma membrane. (SP method, high power microscopic view).

The expression of β1 integrin was also evaluated in 67 patients with DCIS. The average age of patients included in this study was 51 years old (range 23–84 years). Forty-two cases were presented as multifocal and the architectural pattern was mostly solid, comedo and cribriforme, and in our casuistic, 42 cases was associated with invasive carcinoma. Representative samples of IDC and DCIS are shown in Figure 4. There was no significant relation between the expression of the β1 integrin in DCIS with clinical and pathological factors of prognostic significance. Twenty-two patients were positive for β1 integrin where 10 were pre-menopausal and 12 were post-menopausal. Nineteen presented high nuclear grade and only three patients had low nuclear grade.

The relation between the expression of biomarkers, such as ER and PR, and β1 integrin in IDC and DCIS were not significant (Table 2).

**Table 2:** Frequency of β1 integrin (β1), Progesterone Receptor (PR) and Estrogen Receptor (ER) expression in breast carcinomas

**Markers**		**Group**		***P*-value**
		**IDC**	**DCIS**	
		**n (%)**	**n (%)**	
**β1**	Negative	151 (67,1)	45 (67,2)	1.000^a^
	Positive	74 (32,9)	22 (32,8)	
**PR**	Negative	87 (38,7)	20 (29,9)	0.198^a^
	Positive	138 (61,3)	47 (70,1)	
**ER**	Negative	76 (33,8)	17 (25,4)	0.233^a^
	Positive	149 (66,2)	50 (74,6)	

## Discussion

According to Kononen and colleagues [29] tissue microarrays (TMAs) were developed to allow high throughput analysis of protein expression in tumors tissues and many authors agree that they can be used to validate biomarkers [30-32]. Here, β1 integrin expression and its relationship with survival of patients with IDC were analyzed in a tissue microarray.

During the last years a number of reports have linked integrins to tumorigenicity ranging from local tumor growth to metastasis [33] and it is well documented that integrins contribute to migration and invasion of cancer cells [34]. β1 integrin is the most widely expressed integrin in cells and has been suggested to play a role in predicting the clinical course and prognosis of several types of cancers [20]. However, Brakebusch and colleagues [35] argue that it is not surprising that some studies link tumorigenicity to β1 integrin in the form of down- or up-regulated expression. For example, according to Guo and colleagues [36] β1 integrin is the most abundantly expressed integrin in Non-small-cell lung carcinoma and other authors [37] founded that its expression has been associated with lymph node metastasis in the same type of cancer. Although Kren and colleagues [38] noted that the low expression of β1 integrin promoted tumor cells dissemination in a mouse model of pancreatic β cell carcinogenesis.

In regard to breast cancer, some studies have reported that decreased β1 integrin expression was associated with characteristics of more aggressive disease [39,40], while others observed that the high expression of β1 integrin is associated with decrease survival [20]. Berry and colleagues [41] and Petricevic and co-workers [21] verified no significant correlation with β1 integrin expression and survival of patients with breast carcinoma. Then, it is possible to realize that there are controversies in the studies concerning the expression of this protein as well as its relation with survival rate in patients with breast cancer.

We choose the same score used by Yao and colleagues [20] based on the level of signal and percentage of tumor cells expressing the signal. Here, we provide evidences that β1 integrin high expression (2+ and 3+) can be predictive of response and has impact on specific-free survival in patients with breast cancer. Ours findings did not relate β1 integrin with metastasis free survival. Some authors suggested that β1 integrin is important but not essential for metastasis [35]. All these findings are in accordance with previous reports in different types of cancer [42-44], including previous studies that showed that β1 integrin inhibition induces apoptosis in breast cancer cells [45].

The basement membranes are reservoirs for growth factors [46] and it is well known that specific integrins can activate specific FGRs [11]. For example, Hayashida and colleagues [47] founded that β1 integrin are dependently linked with TGF-β1, and that knocking β1 integrin down enhances the cell collagen production trough TGF-β1. Nowadays, it is well known that the interaction between FGRs and integrins can regulate cell survival and proliferation and supporting tumor growth [48,49].

Some studies linked β1 integrin with members of the FGR family including VEGF [50]. This can promote angiogenesis through up-regulation and/or activation of integrins [51]. Other study demonstrated that VEGF activity is dependent on β1 integrin function. In this study the authors founded that the knockout of β1 integrin in embryonic stem cells, neither the proliferation of the endothelial cells nor sprouting of blood vessels occurred, suggesting that VEGF had no effect in β1-null embryoid bodies [52]. More interestingly Lee and colleagues [51] with *in vitro* studies, with human brain microvascular endothelial cells, showed that blocking β1 integrin, all processes of angiogenesis was inhibited (adhesion, migration, and capillary morphogenesis) and they also suggested that the α6β1 integrin is closely related to the metastasis of breast cancer cells to the brain.

Studies have demonstrated that some oncogenes require specific integrins for tumorigenicity. Integrins are not oncogenic molecules, but some of them can cooperate with oncogene to initiate growth, invasion and progression of the cancer [11]. In a transgenic mouse model of human breast cancer some authors founded that β1 integrin mediates the initiation of mammary tumorigenesis that is driven by the polyoma middle T oncoprotein [53].

Recent data suggest a relationship between HER-2 and β1 integrin. Shimizu and colleagues [54], in a study with breast cancer cell line, suggested that the α6β1 integrin inhibits HER-2 signals by proteolytic cleavage of the cytoplasmic domain of HER-2 and this could also contribute to the regulation of tumor growth. Other authors [55] demonstrated that even under adverse conditions such as hypoxia and chemotherapeutic treatments there is a strong regulation between HER-2 signaling stimulating the expression of the integrin α5 and β1 which promotes tumor cell survival.

In the present study, we found a relationship between low expression of β1 integrin and negativity for HER-2 demonstrating some evidence that this subgroup of patients might have a less aggressive phenotype. Besides, we showed that patients who had high β1 integrin expression showed the poor prognostic.

Angiogenesis is induced by VEGF through its interaction with receptors expressed primarily on the vascular endothelial cell membrane [18] and is well known that tumors depend largely on effective angiogenesis [35]. The amplification of the proto-oncogene HER-2 is observed in approximately 15–30% of all breast cancer samples and has been correlated with a shorter survival [23,56]. An important aspect of the involvement of β1 integrin in angiogenesis and tumorigenicity is the potential implication for tumor treatment [57]. This study shows that β1 integrin expression on tumor cells actually promote tumor progression and acts as a tumor enhancer. In addition, our results indicate that both expression of the β1 integrin and its association with HER-2 and VEGF may be useful in targeted therapies for patients with breast cancer.

One of the main focuses concerning breast cancer has been the identification of the molecular alterations associated with the different stages of the progression disease. According to Bombonati and Sgroi [7] the current model of human breast cancer progression proposes a linear multi-step process which initiates as flat epithelial atypia, progresses to atypical ductal hyperplasia, evolves into DCIS and culminates in the potentially lethal stage of IDC. In our study we do not found association with the expression of β1 integrin in IDC and DCIS. 67,1% of the IDC cases were negative for β1 integrin and 67,2% were negative in DCIS cases, with no significant relation probably due to the limited number of cases.

## Conclusions

Subgroups of patients with negativity for β1 integrin and HER-2 might have a less aggressive phenotype. Taken together with the differential expression of VEGF these findings may be useful in targeted therapies for patients with breast cancer. Although there was no association between β1 integrin expression in IDC and DCIS the relationship in these types of cancer needs to be better understood, and further studies are needed to clarify the molecular basis involved in this process.

## Competing interests

The authors declare that they have no competing interests.

## Authors’ contributions

PBS: participated in conception and design, acquisition of data, analysis and interpretation of data, carry out all of the experiments; JSZ: participated in analysis and interpretation of data, carry out part of the experiments; ARS: participated in study design and coordination, and revising it critically for important intellectual content. EICB: help in material support for obtained and funding, and supervised study. All authors read and approved the final manuscript.
